# Editorial: Multilevel Organization and Functional Integration in Organisms

**DOI:** 10.3389/fphys.2021.626977

**Published:** 2021-01-28

**Authors:** Etienne Roux, Marko Marhl, Matteo Mossio

**Affiliations:** ^1^Univ. Bordeaux, INSERM, Biologie des Maladies Cardiovasculaires, Pessac, France; ^2^UMR8590 Institut d'Histoire et de Philosophie des Sciences et des Techniques, CNRS/Université Paris 1, Paris, France; ^3^Faculty of Medicine, University of Maribor, Maribor, Slovenia; ^4^Faculty of Natural Sciences and Mathematics, University of Maribor, Maribor, Slovenia; ^5^Faculty of Education, University of Maribor, Maribor, Slovenia

**Keywords:** structure, network, function, self-organization, systems

Organisms are self-maintained and self-regulated entities, hierarchically organized at different levels of complexity. The investigation and understanding of multilevel organization and functional integration in organisms require not only experimental work but also biophysical and computational models. Moreover, while additional knowledge is being continuously obtained, many theoretical and philosophical issues are still open. This Research Topic proposes a multidisciplinary approach to these issues, with contributions from biologists, physicists, and philosophers of biology. They cover a broad range of topics, such as the origin of life, the emergence of animal multicellularity and its organizational integration, the existence of physiological regulations, as well as the more general question of causality in biology.

In the origins of life, biogenesis is the progressive constitution of spatial and temporal systems beyond individual proto-metabolic organizations, which requires a minimal form of reproduction. Moreno proposes a conceptual analysis of how prebiotic evolution can be viewed as the emergence of autocatalytic reaction loops between molecules leading to compartmentalized self-maintained and self-reproducing networks. This corresponds to a new form of circular causality, which integrates individual organisms and ecosystems at different spatial and temporal scales. In this view, the notion of biological type is not purely abstract but corresponds to the self-maintenance of an organization through space and time including intergenerational continuity and similarity by iteration of reproducing cycles.

The emergence of animal multicellularity from unicellular organisms is an important issue in evolutionary biology. According to Newman, the origin of the structural motifs of animal morphology such as multilayered, hollow, elongated structures, etc., is the consequence of fundamental cellular functions (contraction, excitability…) that were present in ancestral unicellular organisms, combined with the generic physical forces acting in mesoscale aggregate of cells (10^−3^–10^−2^m). Combination of mesoscale physics and genetic toolkits such as morphogens that mediate cell association and behavior generate reproducible dynamical patterning modules responsible for the organizational properties of animals. The emergence of Metazoan morphology can hence be understood as the consequence of physico-genetic effects specific to the multicellular context. Animals, in most of the cases large free-moving entities, are characterized by a specific kind of multicellularity. Arnellos and Keijzer propose a theoretical approach of bodily complexity in animals grounded on transitions from cilia-based to contraction/muscle-based motility, in which muscle-based and myoepithelial-based tissue contraction plays a critical role. The subsequent paper addresses the fact that multicellularity results not only from the spatial organization of cells but also from the organization of the intercellular space itself. Developing the concept of spatial control, Bich, Pradeu et al. analyze how the extracellular space organization, particularly the extracellular matrix (ECM), controls cell differentiation, and the emergence of some specialized functions like immunity. The authors also explain how the notion of spatial control is useful for understanding aging and cancer. The importance of the interactions between the ECM and different cell types in cancer development is evidenced by Pally et al. Combining 3D experimental and computational approaches, the authors show how the interplay between the ECM, the malignant epithelial cells, and the unaffected stroma cells is responsible for the remodeling processes that determine the progression of breast carcinomatosis.

Organisms are characterized by the existence of multiple regulatory processes that are critical for their self-maintenance. One of the most well-known and studied phenomena is the endocrine regulation of glycemia by the pancreatic hormones. In beta-cells of the pancreatic islets, insulin release is triggered by the calcium signal, whose critical determinants include the spatial and temporal interactions among the cells. To grasp the complexity of the calcium signals recorded on isolated pancreatic islets, Korošak and Rupnik have used the Random Matrix Theory (RMT), a fitting mathematical framework, to separate cell-cell interactions happening by chance from those occurring by specific interactions. Considering the same biological system, StoŽer et al. have developed a computational model of interconnected beta cells predicting the calcium signaling in pancreatic islets. The model highlights the importance of the cellular network's heterogeneity and the time lag in the cell-cell interactions generating an integrated calcium signal. Insulin release by pancreatic islets depends on the self-organized critical dynamics of the beta cell networks, which provides a well-controlled glycemia. In their conceptual analysis, Bich, Mossio et al. argue for the abandon of the classical view and language of glycemia regulation in terms of feedback loops centered on set-point and error monitoring. Instead, they propose a theoretical framework according to which glycemia regulation is the consequence of the mutual dependence among a set of functional structures acting as constraints, the so-called organizational closure. Another example of physiological regulation is the sensing by the endothelial cells of the shear stress exerted by the blood flow on the walls of the vessels (WSS). Roux et al. argue that cellular tensional pre-stress, or biotensegrity, is a relevant conceptual framework to understand how endothelial cells are sensitive to the spatial and temporal characteristics of the WSS. By its consequence on blood flow via vascular morphogenesis and remodeling and vasoreactivity, WSS sensing generates a local-global causal loop that determines the ability of the vascular system to ensure the perfusion of the tissues through the maintenance of stable local WSS value. The authors show that the classical set point theory is unable to account for these regulatory processes that should be viewed as dynamical, and not algorithmic, ones acting in a self-organized way.

Ellis and Kopel argue for a fundamental difference in causation between physical vs. biological processes. Though grounded on physical processes, biological systems share some specific properties such as goal-directedness, organization, and information flow. These properties are linked through a specific kind of branching causation. In quantum physics, branching causation is related with the irreducible randomness of quantum outcomes, whereas in biology it corresponds to a branching logic operating at each hierarchical level of biological organization. Using the example of ion channels, the authors argue that a digital logic of ON and OFF processes at the biomolecular level underlies the emergence of macroscale branching dynamics. Noble et al. develop and specify the concept of biological relativity, according to which there is no privileged level of causality in biology. The authors argue that biological systems are characterized by a multiscale network of interactions, in which any part of the network may affect every other part. The authors defend the concept of conditioned causation, i.e., a state of a system where it would be misleading to attribute causation to any particular element. In biological systems, causal loops are at play between different levels of organization, even though they are asymmetric. Upward causation is the fact that lower interacting elements produce change at higher levels, whereas downward causation, or downward determination, is the sets of initial and boundary conditions imposed by higher levels of organization. According to Bizzarri et al., the fact that organisms are complex systems requires a specific conceptual framework for the investigation and explanation of biological systems. In biology, phenomena are processes rather than material objects, meaning that biological entities have relational properties only. The authors propose a “mesoscopic way of thinking” to understand the emergence of relational organizations, their self-maintenance, and, as in pathology, their possible collapse. At the mesoscopic levels, the stochastic fluctuations that characterize the microscopic level turn into ordered behavior and the emergence of regularities in the reciprocal interactions of the parts.

## Author Contributions

All authors listed have made a substantial, direct and intellectual contribution to the work, and approved it for publication.

## Conflict of Interest

The authors declare that the research was conducted in the absence of any commercial or financial relationships that could be construed as a potential conflict of interest.

